# Evaluation of Pharmacological and Phytochemical Profiles of *Piptadeniastrum africanum* (Hook.f.) Brenan Stem Bark Extracts

**DOI:** 10.3390/biom10040516

**Published:** 2020-03-28

**Authors:** Kouadio Ibrahime Sinan, Annalisa Chiavaroli, Giustino Orlando, Kouadio Bene, Gokhan Zengin, Zoltán Cziáky, József Jekő, Mohamad Fawzi Mahomoodally, Marie Carene Nancy Picot-Allain, Luigi Menghini, Lucia Recinella, Luigi Brunetti, Sheila Leone, Maria Chiara Ciferri, Simonetta Di Simone, Claudio Ferrante

**Affiliations:** 1 Department of Biology, Science Faculty, Selcuk University, Campus, Konya, Konya 42130, Turkey; sinankouadio@gmail.com; 2 Department of Pharmacy, “G. d’Annunzio” University Chieti-Pescara, 66100 Chieti, Italy; annalisa.chiavaroli@unich.it (A.C.); luigi.menghini@unich.it (L.M.); lucia.recinella@unich.it (L.R.); uigi.brunetti@unich.it (L.B.); sheila.leone@unich.it (S.L.); mariachiara.ciferri@outlook.it (M.C.C.); disimonesimonetta@gmail.com (S.D.S.); claudio.ferrante@unich.it (C.F.); 3 Laboratoire de Botanique et Phytothérapie, Unité de Formation et de Recherche Sciences de la Nature, 02 BP 801 Abidjan 02, Université Nangui Abrogoua, Abidjan 00225, Ivory Coast; kouadio777@gmail.com; 4 Agricultural and Molecular Research and Service Institute, University of Nyíregyháza, 4400 Nyíregyháza, Hungary; cziaky.zoltan@nye.hu (Z.C.); jjozsi@gmail.com (J.J.); 5 Institute of Research and Development, Duy Tan University, Da Nang 550000, Vietnam; f.mahomoodally@uom.ac.mu; 6 Department of Health Sciences, Faculty of Science, University of Mauritius, Réduit 230, Mauritius; picotcarene@yahoo.com

**Keywords:** medicinal plants, bioactive compounds, hyperpigmentation, diabetes, dopamine

## Abstract

The stem bark (SB) of *Piptadeniastrum africanum* (PA) has been extensively used in African traditional medicinal systems. However, there is a dearth of scientific information regarding its possible activity in the management of type II diabetes, Alzheimer’s disease, and skin hyperpigmentation disorders. This study therefore attempted to elucidate the in vitro inhibitory action of ethyl acetate, methanol, and water extracts of *P. africanum* stem bark (PA-SB) on α-amylase, α-glucosidase, acetylcholinesterase, butyrylcholinesterase, and tyrosinase. Cell viability, catecholamine, and 3-hydroxykynurenine levels of hypothalamic HypoE22 cells exposed to PA-SB extracts were also investigated. The phytochemical profiles of the extracts were determined by high performance liquid chromatography (HPLC) and antioxidant properties were investigated. Saponin (867.42 mg quillaja equivalent/g) and tannin (33.81 mg catechin equivalent/g) contents were higher in the methanol extract. Multiple dihydroxy-trimethoxy(iso)flavone isomers, loliolide, eriodictyol, naringenin, luteolin, chrysoeriol, apigenin, and liquiritigenin, were characterized from PA-SB extracts using HPLC. The methanol extract of PA-SB showed highest inhibitory activity against acetylcholinesterase (4.88 mg galantamine equivalent (GALAE)/g extract), butyrylcholinesterase (5.37 mg GALAE/g extract), and tyrosinase (154.86 mg kojic acid equivalent/g extract) while α-glucosidase was effectively inhibited by the ethyl acetate extract (15.22 mmol acarbose equivalent/g extract). The methanol extract of PA-SB also showed potent antioxidant properties (493.87, 818.12, 953.07, and 732.19 mg Trolox equivalent/g extract, for 1,1-diphenyl-2-picrylhydrazyl (DPPH), 2,2′-azino-bis(3-ethylbenzothiazoline-6-sulphonic acid (ABTS), cupric reducing antioxidant capacity (CUPRAC), and ferric reducing antioxidant power (FRAP) assays, respectively). PA-SB extracts exhibited antioxidant activity and promising inhibition against key enzymes related to type II diabetes, Alzheimer’s disease, and skin hyperpigmentation disorders. Additionally, all extracts were able to contrast hydrogen peroxide-induced oxidative stress, in HypoE22 cells, thus restoring basal catecholamine and 3-hydroxykinurenine levels, whereas only methanol and water extracts stimulated basal dopamine release. Overall, data from the present study contribute to the biological assessment of *P. africanum* that appears to be a promising source of natural compounds with protective and neuromodulatory effects.

## 1. Introduction

*Piptadeniastrum africanum* (Hook.f.) Brenan (Mimosaceae), a massive tree which can reach up to 50 m, is widely distributed across Western Africa tropical rainforests of Angola, Uganda, Senegal Cameroon, Democratic Republic of Congo, Nigeria, and Sudan [1]. *P. africanum* has been extensively used in African traditional medicinal systems. In Uganda, the stem bark of *P. africanum* has been utilized by traditional medicine practitioners for the treatment of tuberculosis and HIV/AIDS [2]. Pygmies Baka, traditional healers from Cameroon, used *P. africanum* stem bark to treat abdominal pain, fever, and cough; leaves and fruits are used as aphrodisiac, tonic, abortifacient, and to treat oedema urethritis [3]. *P. africanum* was also reported to be used in the management of constipation, anaemia, lumbago, meningitis, edema, rheumatism, convulsion, gastric ulcer, and for wound healing. A decoction of *P. africanum* stem bark was used against sexual asthenia, constipation, intestinal cramps, and back pain among the Nkundo people, from the Democratic Republic of Congo [4]. Dlamini and colleagues (2019) summarized the ethnomedicinal uses of *P. africanum* across different countries in Africa. As such, they reported that in Nigeria the stem bark and root were used to treat sickle cell anemia and cancer, respectively; in Guinea the stem bark is used for its antibacterial properties, whereas leaves are used in the management of diabetes; in Ghana, Benin, and Gabon the bark is used for waist pain impotence [1].

Several studies have explored the pharmacological potential of *P. africanum*. Mbiantcha and co-workers have reported the antiarthritic activity of methanol and aqueous extracts of *P. africanum* stem bark in Freund’s adjuvant induced arthritis rat model. The researchers claimed that the observed antiarthritic activity could be explained by the in vitro activity of extracts on the immune system as well as the inhibition of the release of pro-inflammatory mediators, namely, TNF-α and IL-1β [4]. Antifungal activity (*Pyricularia grisea*) was observed for aqueous and methanol extracts of *P. africanum* stem bark. Isolation of tannin and saponin from *P. africanum* stem bark methanol extract revealed promising activity against five different *Pyricularia grisea* strains [3]. The aqueous extract of *P. africanum* stem bark exhibited pronounced antiprotozoal activity against *Trypanosoma b. brucei* (IC_50_ 7.94 μg/mL), *Trypanosoma cruzi* (IC_50_ 6.20 μg/mL), *Leishmania infantum* (IC_50_ 6.01 μg/mL), and *Plasmodium falciparum* chloroquine-and pyrimethamine-resistant K1 strain (IC_50_ 6.11 μg/mL). In addition, *P. africanum* stem bark aqueous extract (CC_50_ 8.80 μg/mL) showed cytotoxicity against secondary human lung fibroblasts, MRC-5 cells [5]. In line with tradition use of *P. africanum* to treat gastric ulcer, in vivo investigation revealed that aqueous and methanol extracts of *P. africanum* stem bark significantly (*p* < 0.01) inhibited gastric ulceration induced by HCl/ethanol (81.38; 98.75, and 100% for the aqueous extract and 75.83, 89.76, and 96.52% for the methanol extract). Besides, preventive and curative effects were observed and might be related to the presence of alkaloids, flavonoids, phenols, and saponins [6]. For example, Brusotti et al. [3] reported a good fungicidal effect of saponin fraction from *P. africanum* stem bark. In addition, Note et al. [7] found a new saponin (piptadeniaoside) and two known saponins from stem bark.

Reports of previous studies have demonstrated the potential of *P. africanum*. From our literature search it appeared that no study has been undertaken to elucidate the possible inhibitory ability of *P. africanum* stem bark on enzymes involved in the pathogenesis of complications such as type II diabetes, Alzheimer’s disease, and skin hyperpigmentation disorders. Hence, the present study focuses on the in vitro evaluation of the enzyme inhibitory activity of the ethyl acetate, methanol, and water extracts of *P. africanum* stem bark against α-amylase, α-glucosidase, acetylcholinesterase (AChE), butyrylcholinesterase (BChE), and tyrosinase. With regard to the role of oxidative stress in the pathogenesis of type II diabetes, Alzheimer’s disease, and skin hyperpigmentation disorders, the antioxidant activity of the extracts will also be assessed using different standardized in vitro bioassays. Besides, the phytochemical profile of the extracts was also reported. Finally, extract neuromodulatory effects were investigated on hypothalamic neurotransmitters, namely norepinephrine (NE) and dopamine (DA), involved in feeding and metabolism [8,9]. Specifically, the extracellular levels of DA and NE were measured in hypothalamic HypoE22 cells stimulated with extracts, either in basal or hydrogen peroxide-induced oxidative conditions. In HypoE22 cells, we also investigated extract effects on extracellular levels of 3-hydroxykinurenine (3-HK), a recognized marker of neurotoxicity [10].

## 2. Materials and Methods

### 2.1. Sample Collection and Extraction

Plant samples were collected in Ivory Coast (Agboville, Region of Agnéby-Tiassa) in 2019 (at flowering season). Taxonomical identification was performed by Kouadio Bene, a botanist. The stem barks were selected for extraction and dried at room temperature (about 10 days in shade). Then, the plant samples were powdered by using a laboratory mill.

To prepare extracts, we selected maceration technique for ethyl acetate (EA) and methanol. For this purpose, powdered plant materials (10 g) was mixed with these solvents (200 mL) at room temperature for 24 h. Subsequently, the extracts were concentrated by using a rotary-evaporator under vacuum. For water extract, the infusion was prepared (5 g of samples were kept in 100 mL of boiled water for 20 min) and then was lyophilized. All extracts were stored at +4 °C until analyses. The extraction yields are given in Table 1.

### 2.2. Chemical Profiling

Total flavonoid, phenol, phenolic acid, flavanol, tannin, and saponin contents of extracts were determined as previously described [11]. Results were expressed as equivalent of standard compounds, such as rutin (mg RE/g) for flavonoids, gallic acid (mg GAE/g) for phenols, caffeic acid (mg CAE/g) for phenolic acids, catechin (mg CE/g) for flavanols and tannins, and quillaja (mg QE/g) for saponins. Extract bioactive profile was determined using a Dionex Ultimate 3000RS UHPLC instrument. A Thermo Accucore C18 (100 mm × 2.1, mm i. d., 2.6 μm) column was used to separate the compound in the extracts [12]. Details about analytical and chromatographic parameters are given in Tables S1–S3 (supplementary material and methods available online). In addition, total ion chromatograms were depicted in Figures S1–S3 (supplementary material and methods file, available online).

### 2.3. Determination of Antioxidant and Enzyme Inhibitory Effects

Scavenging/reducing and chelating properties of the extracts were assessed through colorimetric metal chelating, phosphomolybdenum, ferric reducing antioxidant power (FRAP), cupric reducing antioxidant capacity (CUPRAC), 2,2′-azino-bis(3-ethylbenzothiazoline-6-sulphonic acid (ABTS), 1,1-diphenyl-2-picrylhydrazyl (DPPH), ferrozine, and phosphomolybdenum assays (Grochowski et al., 2017). Furthermore, enzyme inhibitory effects of each extract were determined against cholinesterases (by Ellman’s method), tyrosinase, α-amylase, and α-glucosidase, as previously described (Zengin et al., 2017).

### 2.4. Artemia Salina Lethality Bioassay

*Artemia salina* lethality bioassay was performed as previously reported [13]. Briefly, brine shrimp larvae were bred at 25–28 °C for 24h in presence of *P. africanum* extracts (10–10,000 µg/mL) dissolved in incubation medium (artificial sea water). The extract-induced lethality was expressed as LC_50_ values.

### 2.5. In Vitro Studies

Rat hypothalamic Hypo-E22 cells were cultured in DMEM supplemented with 10% (*v*/*v*) heat-inactivated fetal bovine serum and penicillin-streptomycin (100 μg/mL). Cells were seeded into 96-well plates at 5 × 10^3^ cells/well and grown at 37 °C in a humified atmosphere of 5% CO_2_. Cell viability was evaluated after 24 h of extract stimulation (0.1–10 µg/mL) by MTT (3-(4,5-dimethyl-thiazol-2-yl-]-2,5-diphenyl tetrazolium bromide) growth assay [14,15,16].

For pharmacological evaluations, cells were stimulated with extracts for 24 h as well. In order to test extract efficacy against oxidative stress, cells were challenged with 1 mM hydrogen peroxide for 1 h, after pre-treatment with extracts for 24 h. Finally, supernatants were collected and stored at −20 °C.

### 2.6. High Performance Liquid Chromatography (HPLC) Determination of Dopamine (DA), Norepinephrine (NE), and 3-Hydroxykinurenine (3-HK)

Extracellular DA, NE, and 3-HK levels were analyzed by HPLC (PU-2080 chromatographic pump; Jasco, Tokyo, Japan) coupled to ESA (Chelmsford, MA, USA) Coulochem III coulometric detector, equipped with a microdialysis cell (ESA-5014b). The analytical conditions were selected according to previous studies [17,18].

### 2.7. Statistical Analysis

Results were reported as means ± standard deviation. The statistical evaluations were performed by R 3.6.2. One-way analysis of variance (ANOVA), followed by Turkey’s multiple range test at *p* < 0.05 level was employed to assess the significant difference among the biological activities of the samples. Venn diagram analysis of identified phytochemicals was done with the online tool [19]. The pharmacological properties were analyzed by ANOVA, followed by Newman–Keuls comparison multiple test. These experiments were the mean ± standard deviation of three experiments performed in quadruplicate. Statistical significance was set at *p* < 0.05.

## 3. Results and Discussion

Biological activity associated to plants result from the interaction of phytochemicals with specific metabolic pathways. General phytochemical analysis provides an insight of the possible activity of specific classes of phytochemicals within a plant extract. The analysis of *P. africanum* stem bark showed that the methanol and water extracts were rich in phenolic, phenolic acids, and tannins (Table 1). The saponin content was found to be higher in the methanol extract (867.42 mg QE/g). It was also noted that the methanol extract (33.81 mg CE/g) contained higher amounts of tannin compared with the ethyl acetate and water extracts (7.99 and 23.84 mg CE/g, respectively).

**Figure 1:**
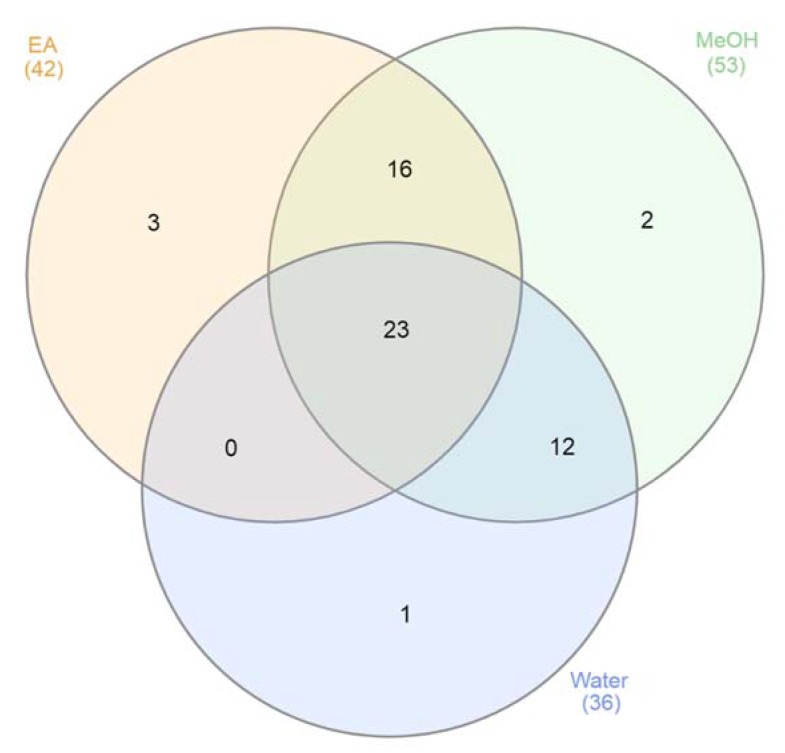
Venn diagram of identified compound numbers in all tested extracts.

Further phytochemical screening was performed using the HPLC technique. Data for ethyl acetate, methanol, and water extracts were presented in Table 2. About 42, 53, and 36 compounds were tentatively identified from ethyl acetate, methanol, and water extracts, respectively (Figure 1). Piptadenin, having a precursor at 485.3267 (M − H)^−^ (this study), was previously isolated from *P. africanum* stem bark methanol extract [20]. β-Sitostenone showing fragment ions at *m*/*z* 395.3688, 255.2114, 123.0809, 109.0654, and 97.0655 was identified in the methanol and ethyl acetate extracts of *P. africanum* stem bark. Loliolide, eriodictyol, naringenin, luteolin, chrysoeriol, apigenin, liquiritigenin, and multiple dihydroxy-trimethoxy(iso)flavone isomers were identified from *P. africanum* stem bark extracts.

Clinical data attest that over the past decades there has been an exponential increase in the incidence of Alzheimer’s disease. These findings have fostered the need for further in-depth research to attempt to elucidate the cause and lifestyle patterns related to Alzheimer’s disease. In this sense, a growing body of research has indicated that inflammation, tau hyperphosphorylation, amyloid plaques formation, and apolipoprotein E4 function showed intertwined relationships in the progression of both type II diabetes and Alzheimer’s disease. In this regard, the development of biologically active agents capable of managing both conditions are considered as an interesting treatment modality. Enzymes have been and are still key targets in the management of type II diabetes and Alzheimer’s disease. Therefore, the present study set out to investigate the possible inhibitory activity of *P. africanum* stem bark extracts on AChE and BChE (targets in Alzheimer’s disease management) and α-amylase and α-glucosidase (targets in type II diabetes management). As shown in Table 3, the methanol extract of *P. africanum* stem bark showed the highest activity against both AChE (4.88 mg GALAE/g extract) and BChE (5.37 mg GALAE/g extract). Loliolide ((M + H)^+^ 197.1178), identified from the extracts of *P. africanum* stem bark, was reported to be previously isolated from *Gloiopeltis furcate* and exhibited AChE inhibitory activity (IC_50_ 7.57 µg/mL). Naringenin derivatives were characterized from the stem bark extracts of *P. africanum*. Interestingly, naringenin attenuated cognitive deficit in scopolamine-induced amnesia mice [21]. Another study reported that naringenin derivative (8-prenylnaringenin) exhibited micro-molar range inhibition against BChE (IC_50_ 86.58 µM compared with galantamine 46.58 µM), showing hydrogen bonds and π-π stacking interaction with Ser198, Gly117, and His438 in in silico molecular docking studies [22]. Trigonelline, an alkaloid identified at *m*/*z* 138.0555 ((M + H)^+^), inhibited AChE with an IC_50_ value of 233 μM while 1.27 μM was recorded for galantamine [23]. Trigonelline was administered to d-galactose induced cognitive impaired mice and a significant decline in AChE level along with reduced oxidative stress and advanced glycation end products levels were observed [24]. It is important to highlight that the higher inhibitory action of the methanol extract of *P. africanum* stem bark on AChE and BChE might be related to the synergistic action of multiple phytochemicals rather than one single molecule. Data presented in Table 3 revealed that ethyl acetate, methanol, and water extracts of *P. africanum* stem bark were weak α-amylase inhibitors (0.35–0.89 mmol ACAE/g extract). On the other hand, only the ethyl acetate extract (15.22 mmol ACAE/g extract) displayed inhibitory action against α-glucosidase. Trigonelline, identified in the ethyl acetate extract of *P. africanum* stem bark, has been previously reported to inhibit α-glucosidase in diabetic rats [25]. Besides, it can be argued that the observed α-glucosidase inhibition was related to multiple flavonoids present in this extract, such as eriodictyol, naringenin, chrysoeriol, luteolin, and apigenin [26]. Likewise, the observed inhibitory activity might be caused by the concerted synergistic action of several phytochemicals.

The inhibition of *P. africanum* stem bark extracts on tyrosinase was also investigated in the present study and data were reported in Table 3. The inhibitory activity was as follows methanol > ethyl acetate > water extract. Here also, the methanol extract (154.86 mg KAE/g extract) of *P. africanum* stem bark was the most active. The quest for naturally occurring tyrosinase inhibitors stems from the increasing demand for plant-based dermatological products. Among phytochemicals identified from the methanol extract, eriodictyol has been previously reported to reduce tyrosinase activity in cultured murine melanoma B16-F10 cells and also demonstrated cellular antioxidant properties [27]. Eriodictyol (total DNA damage value of 114.5, 116, and 208 for non-treated cell, cells treated with 50 μM eriodictyol, cells treated with hydrogen peroxide) also showed no genotoxicity when tested on primary human keratinocyte cells [27]. A study conducted by Bouzaiene and co-workers highlighted that naringenin caused an increase in tyrosinase activity in B16F10 melanoma cells [28]. It can be argued that other classes of phytochemicals were responsible for the observed tyrosinase inhibition.

Oxidative stress is regarded as a key pathological player that precedes or accompany the pathogenesis of Alzheimer’s disease, type II diabetes, and skin hyperpigmentation problems [29]. Studies have shown that intake of antioxidants could prevent or delay the onset/progress of many diseases [30,31,32]. In order to have a comprehensive understanding of the antioxidant property of *P. africanum* stem bark extracts, multiple assays were employed (Table 4). Evaluation of the total antioxidant capacity of the extracts by phosphomolybdenum method showed that the methanol extract was more active, followed by water and ethyl acetate extracts. The phosphomolybdenum method has been routinely used to assess the total antioxidant capacity of plant extracts, whereby in the presence of antioxidant compounds Mo (VI) is reduced to Mo (V) which is a green colored complex [33]. The high reactivity and relative instability of free radicals are related to their unpaired electron which may damage biological molecules, such as DNA, lipids, carbohydrates, and proteins [34]. In the current work, the radical scavenging property of extracts was assessed using DPPH and ABTS methods. Higher activity was observed for the methanol extract (493.87 and 818.12 mg TE/g extract, for DPPH and ABTS, respectively). Likewise, the methanol extract showed higher reducing activity in CUPRAC (953.07 mg TE/g extract) and FRAP (732.19 mg TE/g extract) assays. Transition metals are also involved in oxidative stress by promoting the formation of hydroxyl radicals via Fenton reactions [35]. It was noted that the water extract (14.21 mg EDTAE/g) showed the highest metal chelating properties.

**Figure 2:**
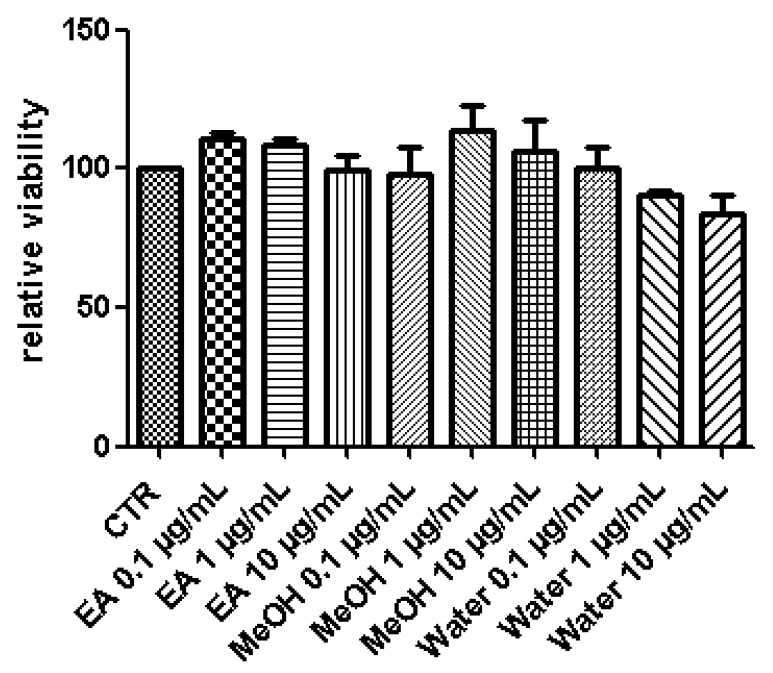
Effects of *P. africanum* water, methanol (MeOH), and ethylacetate (EA) extracts (0.1–10 µg/mL) on hypothalamic HypoE22 cell line viability (MTT test), 24 h after treatment.

**Figure 3:**
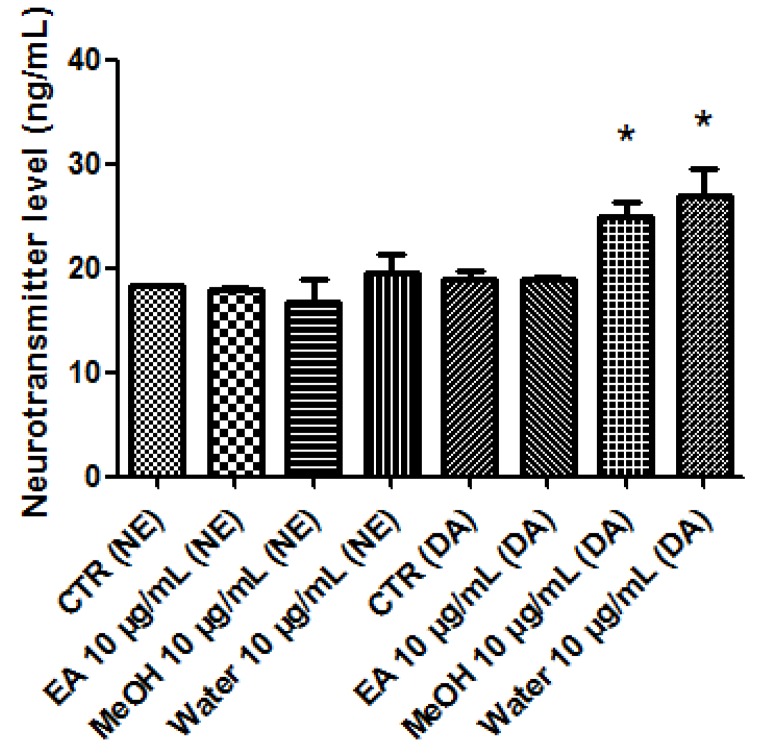
Effects of *P. africanum* water, methanol (MeOH), and ethylacetate (EA) extracts (10 µg/mL) on extracellular levels (ng/mL) of dopamine (DA) and norepinephrine (NE). Neurotransmitters were measured in the medium of hypothalamic HypoE22 cells stimulated with extracts in basal conditions. ANOVA, *p* < 0.05; post hoc test, * *p* < 0.05 vs. CTR group.

**Figure 4:**
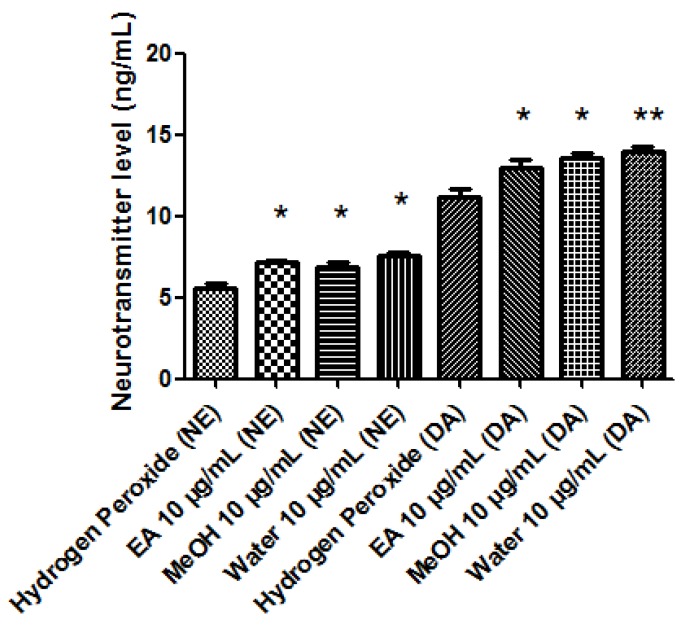
Effects of *P. africanum* water, methanol (MeOH), and ethylacetate (EA) extracts (10 µg/mL) on extracellular levels (ng/mL) of dopamine (DA) and norepinephrine (NE). Neurotransmitters were measured in the medium of hypothalamic HypoE22 cells stimulated with extracts and challenged with hydrogen peroxide (1 mM). ANOVA, *p* < 0.01; post hoc test, * *p* < 0.05, ** *p* < 0.01 vs. Hydrogen Peroxide group.

**Figure 5:**
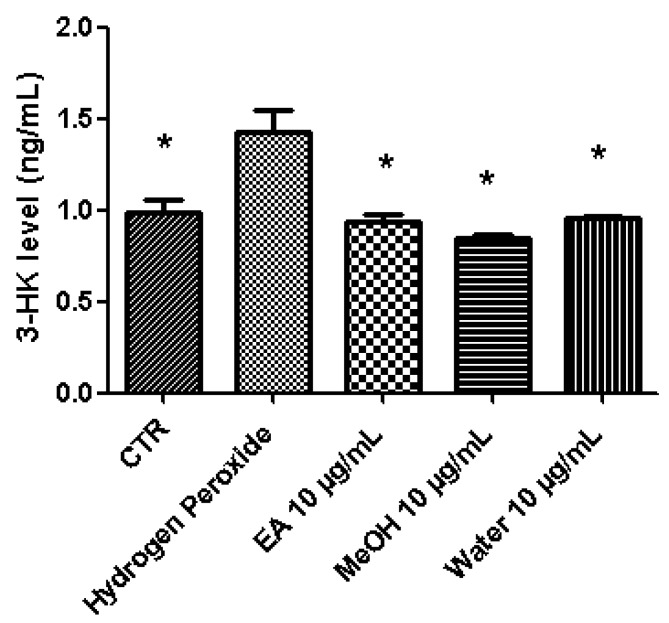
Inhibitory effects induced by *P. africanum* water, methanol (MeOH), and ethylacetate (EA) extracts (10 µg/mL) on extracellular levels (ng/mL) of 3-hydroxykynurenine (3-HK). 3-HK was measured in the medium of hypothalamic HypoE22 cells stimulated with extracts and challenged with hydrogen peroxide (1 mM). ANOVA, *p* < 0.05; post hoc test, * *p* < 0.05 vs. Hydrogen Peroxide group.

A pharmacological investigation was conducted in order to explore protective and neuromodulatory effects induced by *P. africanum* extracts. In this regard, the biocompatibility limit was initially determined through multiple independent methods. The brine shrimp lethality test was performed in order to evaluate extract cytotoxicity. The assay yielded LC_50_ values > 100 µg/mL and based on previous studies [13,36], a concentration at least ten fold lower was considered as biocompatibility limit for the subsequent in vitro tests on hypothalamic HypoE22 cells, that were selected as a model of neuronal cell line responding to neuromodulatory and protective agents [17,35]. In the MTT test, HypoE22 cells were stimulated with the extracts in the range 0.1–10 µg/mL and a null effect on cell viability was observed (Figure 2). This is consistent, at least in part, with previous studies suggesting anti-proliferative effects induced by *P. africanum* extracts at higher concentrations [37,38]. Based on results gathered from the MTT test, a concentration of 10 µg/mL was chosen for evaluating protective and neuromodulatory effects. Extract effects on extracellular neurotransmitter levels were investigated, either in basal or hydrogen peroxide (1 mM)-induced oxidative stress condition. In basal condition, the stimulation of extracellular DA level following water and methanol treatment (Figure 3) suggests a potential influence of these extracts on hypothalamic appetite network underlying feeding behavior [7,8]. Basing on qualitative fingerprint analysis, we hypothesize that the stimulation of DA release induced by methanol and water extracts could be due, at least in part, to the presence of trigonelline, that was found effective in stimulating DA release from pheochromocytoma (PC)12 cells [39]. Considering the scavenging/reducing effects induced by all extracts (Table 4), they were also tested on HypoE22 cells challenged with a well-established oxidative stress stimulus (hydrogen peroxide). The blunting effects induced by extracts on hydrogen peroxide-induced depletion of hypothalamic catecholamines (Figure 4) and up-regulation of 3-HK (Figure 5), a well-known index of neurotoxicity [10], suggest *P. africanum* extracts as promising antioxidant agents. Nevertheless, further in vivo studies need to confirm the efficacy in reducing oxidative stress biomarkers, in the brain. It is reasonable to hypothesize that the effectiveness of all tested extracts in contrasting hydrogen peroxide-induced hypothalamic catecholamine depletion and 3-HK increase might be related to the phenolic compounds present in the extracts (Table 1).

## 4. Conclusions

*P. africanum* has been extensively used in traditional medicine across Africa. Previous studies reported biological properties of *P. africanum*, including antiarthritic, antifungal, and antiprotozoal activities. However, a paucity of scientific information regarding the possible inhibitory activity of *P. africanum* on enzymes related to Alzheimer’s disease, type II diabetes, and skin hyperpigemenation problems were noted. Therefore, the present study emphasized on unravelling the possible action of *P. africanum* stem bark extracts on α-amylase, α-glucosidase, acetylcholinesterase, butyrylcholinesterase, and tyrosinase. The methanol extract proved to be an active inhibitor of acetylcholinesterase, butyrylcholinesterase, and tyrosinase and showed interesting antioxidant properties in radical scavenging and reducing potential assays. Consistent with the scavenging/reducing properties, all extracts were able to contrast hydrogen peroxide-induced catecholamine depletion, in HypoE22 cells, although only methanol and water extracts stimulated basal dopamine release. Overall, data from the present study contribute to the biological assessment of *P. africanum*, that appears to be a promising source of natural compounds with protective e neuromodulatory effects.

## Figures and Tables

**Table 1:** Extraction yields and total bioactive components of the tested samples.

Samples	Extraction Yields (%)	Total Phenolic Content (mg GAE/g Extract)	Total Flavonoid Content (mg RE/g Extract)	Total Phenolic Acid Content (mg CAE/g)	Total Flavanol Content (mg CE/g)	Total Tannin Content (mg CE/g)	Total Saponin Content (mg QE/g)
EA	0.64	56.62 ± 0.19 ^b^	2.95 ± 0.05 ^a^	0.13 ± 0.01 ^c^	2.54 ± 0.04 ^b^	7.99 ± 0.45 ^c^	212.78 ± 11.08 ^c^
MeOH	13.69	203.71 ± 1.61 ^a^	2.47 ± 0.23 ^b^	16.15 ± 1.17 ^b^	8.70 ± 0.15 ^a^	33.81 ± 2.22 ^a^	867.42 ± 5.78 ^a^
Water	16.19	205.33 ± 1.11 ^a^	2.31 ± 0.03 ^c^	40.03 ± 0.74 ^a^	1.93 ± 0.07 ^c^	23.84 ± 2.23 ^b^	675.81 ± 34.88 ^b^

Values expressed are means ± S.D. of three parallel measurements. GAE: Gallic acid equivalent; RE: Rutin equivalent; CE: catechin equivalent; CAE: caffeic acid equivalent; QE: Quillaja equivalent; EA: Ethyl acetate; MeOH: Methanol; nd: not detected. Different superscripts indicate differences in the extracts (*p <* 0.05).

**Table 2:** Chemical composition of the tested extracts.

No.	Name	Formula	Ethyl Acetate	Methanol	Water	Literature
1	Trigonelline	C7H8NO2	+	+	+	
2	Gallocatechin (Casuarin, Gallocatechol)	C15H14O7	−	+	+	
3 ^1^	Catechin	C15H14O6	+	+	+	
4 ^1^	Epigallocatechin (Epigallocatechol)	C15H14O7	−	+	+	
5 ^1^	Vanillin	C8H8O3	+	+	+	
6	Naringenin-6,8-di-*C*-glucoside	C27H32O15	−	+	+	
7	Quercetin-*O*-hexoside	C21H20O12	−	+	+	
8	Loliolide	C11H16O3	+	+	+	
9	Trihydroxystilbene	C14H12O3	−	+	−	
10	Tetrahydroxyxanthone	C13H8O6	−	+	+	
11	Isoliquiritigenin	C15H12O4	+	+	+	
12 ^1^	Eriodictyol (3′,4′,5,7-Tetrahydroxyflavanone)	C15H12O6	+	+	+	
13	Abscisic acid	C15H20O4	−	+	+	
14	Methoxy-pentahydroxy(iso)flavone	C16H12O8	+	+	+	
15	Dihydroxy-methoxy(iso)flavone isomer 1	C16H12O5	−	+	+	
16	Dihydroxyflavone	C15H10O4	+	+	+	
17	Dihydroxy-dimethoxy(iso)flavone isomer 1	C17H14O6	+	+	+	
18 ^1^	Quercetin (3,3′,4′,5,7-Pentahydroxyflavone)	C15H10O7	−	+	+	
19	Methoxy-tetrahydroxy(iso)flavone-*O*-hexoside	C22H22O12	−	+	+	
20 ^1^	Naringenin (4′,5,7-Trihydroxyflavanone)	C15H12O5	+	+	+	
21	Homoeriodictyol (3′-Methoxy-4′,5,7-trihydroxyflavanone)	C16H14O6	−	+	+	
22	Dihydroxy-dimethoxy(iso)flavone isomer 2	C17H14O6	+	+	+	
23 ^1^	Luteolin (3′,4′,5,7-Tetrahydroxyflavone)	C15H10O6	+	+	+	
24	Methoxy-tetrahydroxy(iso)flavone	C16H12O7	+	+	+	
25	Dimethoxy-tetrahydroxy(iso)flavone	C17H14O8	+	+	−	
26	Dihydroxy-dimethoxy(iso)flavone isomer 3	C17H14O6	+	+	−	
27	Dimethoxy-trihydroxy(iso)flavone-*O*-hexoside	C23H24O12	−	+	+	
28 ^1^	Apigenin (4′,5,7-Trihydroxyflavone)	C15H10O5	+	+	−	
29	Chrysoeriol (Scoparol, 3′-Methoxy-4′,5,7-trihydroxyflavone)	C16H12O6	+	+	+	
30	Liquiritigenin (4′,7-Dihydroxyflavanone)	C15H12O4	+	+	+	
31	Methoxy-trihydroxy(iso)flavone isomer 1	C16H12O6	+	+	+	
32	Dimethoxy-trihydroxy(iso)flavone isomer 1	C17H14O7	+	+	+	
33	Dihydroxy-dimethoxy(iso)flavone isomer 4	C17H14O6	+	+	−	
34	Dihydroxy-trimethoxy(iso)flavone isomer 1	C18H16O7	+	+	−	
35	Dihydroxy-tetramethoxy(iso)flavone	C19H18O8	−	+	−	
36	Methoxy-trihydroxy(iso)flavone isomer 2	C16H12O6	+	+	+	
37	Dihydroxy-trimethoxy(iso)flavone isomer 2	C18H16O7	+	+	−	
38	Dimethoxy-trihydroxy(iso)flavone isomer 2	C17H14O7	+	+	+	
39	Dihydroxy-trimethoxy(iso)flavone isomer 3	C18H16O7	+	+	−	
40	Dihydroxy-dimethoxy(iso)flavone isomer 5	C17H14O6	+	+	−	
41	Dihydroxy-methoxy(iso)flavone isomer 2	C16H12O5	−	+	+	
42	Dihydroxy-trimethoxy(iso)flavone isomer 4	C18H16O7	+	+	−	
43	Dihydroxy-dimethoxy(iso)flavone isomer 6	C17H14O6	+	+	−	
44	Dihydroxy-trimethoxy(iso)flavone isomer 5	C18H16O7	+	+	−	
45	Bruguierol A	C12H14O2	+	+	−	
46	Dihydropiptadenin or isomer	C30H48O5	+	+	+	
47	Hexadecanedioic acid	C16H30O4	+	+	+	
48	Piptadenin	C30H46O5	+	+	−	[20]
49	Tetrahydropiptadenin or isomer	C30H50O5	+	+	+	
50	Hydroxyhexadecanoic acid	C16H32O3	+	+	+	
51	22β-Hydroxyoleanic acid	C30H48O4	+	+	−	[20]
52	5α-Stigmast-7,22-dien-3-one	C29H46O	+	+	−	[20]
53	β-Sitostenone	C29H48O	+	+	−	
54	Emodin	C15H10O5	−	−	+	
55	Di-*O*-methylellagic acid-*O*-pentoside	C21H18O12	+	−	−	
56	3,3′-Di-*O*-methylellagic acid	C16H10O8	+	−	−	
57	3,3′,4-Tri-*O*-methylflavellagic acid	C17H12O9	+	−	−	

^1^ Confirmed by standard. +: present; −: absent.

**Table 3:** Enzyme inhibitory properties of the tested extracts.

Samples	AChE (mg GALAE/g Extract)	BChE (mg GALAE/g Extract)	α-Amylase (mmol ACAE/g Extract)	α-Glucosidase (mmol ACAE/g Extract)	Tyrosinase (mg KAE/g Extract)
EA	4.37 ± 0.04 ^b^	3.94 ± 0.25 ^c^	0.89 ± 0.04 ^a^	15.22 ± 0.16	134.24 ± 0.76 ^b^
MeOH	4.88 ± 0.09 ^a^	5.37 ± 0.10 ^a^	0.81 ± 0.03 ^b^	na	154.86 ± 0.23 ^a^
Water	4.31 ± 0.02 ^b^	4.77 ± 0.11 ^b^	0.35 ± 0.02 ^c^	na	128.47 ± 0.75 ^c^

Values expressed are means ± S.D. of three parallel measurements. GALAE: Galatamine equivalent; KAE: Kojic acid equivalent; ACAE: Acarbose equivalent; na: not active; EA: Ethyl acetate; MeOH: Methanol. Different superscripts indicate differences in the extracts (*p* < 0.05).

**Table 4:** Antioxidant activities of the tested samples.

Samples	Phosphomolybdenum (mmol TE/g)	DPPH (mg TE/g Extract)	ABTS (mg TE/g Extract)	CUPRAC (mg TE/g Extract)	FRAP (mg TE/g Extract)	Metal Chelating Ability (mg EDTAE/g)
EA	2.07 ± 0.13 ^b^	90.93 ± 0.05 ^c^	139.66 ± 1.73 ^c^	210.26 ± 2.56 ^c^	127.33 ± 0.44 ^c^	na
MeOH	3.93 ± 0.20 ^a^	493.87 ± 1.03 ^a^	818.12 ± 3.68 ^a^	953.07 ± 9.40 ^a^	732.19 ± 22.95 ^b^	10.13 ± 0.40 ^b^
Water	3.78 ± 0.09 ^a^	480.05 ± 0.40 ^b^	558.68 ± 22.89 ^b^	917.88 ± 6.52 ^b^	769.54 ± 7.58 ^a^	14.21 ± 0.52 ^a^

Values expressed are means ± S.D. of three parallel measurements. TE: Trolox equivalent; EDTAE: EDTA equivalent; EA: Ethyl acetate; MeOH: Methanol. Different superscripts indicate differences in the extracts (*p <* 0.05).

## Supplementary Materials

The supplementary material and methods are available online at https://www.mdpi.com/2218-273X/10/4/516/s1. Table S1: Chemical composition of ethyl acetate extract, Table S2: Chemical composition of methanol extract, Table S3: Chemical composition of water extract, Figure S1: Total ion chromatogram of methanol extract in positive (a) and negative (b) ion mode, Figure S2: Total ion chromatogram of ethyl acetate extract in positive (a) and negative (b) ion mode, Figure S3: Total ion chromatogram of water extract in positive (a) and negative (b) ion mode.
