# Supplementary material for: Evaluation of Pharmacological and Phytochemical Profiles of Piptadeniastrum africanum (Hook.f.) Brenan Stem Bark Extracts
Source: Biomolecules. 2020 Mar 28;10(4):516. doi: 10.3390/biom10040516 (PMC7226170; doi:10.3390/biom10040516)
Supplement: Supplementary file 1 [file biomolecules-10-00516-s001.pdf]

Article

Evaluation of Pharmacological and Phytochemical Profiles of *Piptadeniastrum africanum* (Hook.f.) Brenan Stem Bark Extracts

Kouadio Ibrahime Sinan [1], Annalisa Chiavaroli [2], Giustino Orlando [2,*], Kouadio Bene [3], Gokhan Zengin [1,*], Zoltán Cziáky [4], József Jekő [4], Mohamad Fawzi Mahomoodally [5,6], Marie Carene Nancy Picot-Allain [6], Luigi Menghini [2], Lucia Recinella [2], Luigi Brunetti [2], Sheila Leone [2], Maria Chiara Ciferri [2], Simonetta Di Simone [2] and Claudio Ferrante [2]

1 Department of Biology, Science Faculty, Selcuk University, Campus, Konya, 42130 Konya, Turkey; sinankouadio@gmail.com

2 Department of Pharmacy, "G. d'Annunzio" University Chieti-Pescara, 66100 Chieti, Italy; annalisa.chiavaroli@unich.it (A.C.); luigi.menghini@unich.it (L.M.); lucia.recinella@unich.it (L.R.); uigi.brunetti@unich.it (L.B.); sheila.leone@unich.it (S.L.); mariachiara.ciferri@outlook.it (M.C.C.); disimonesimonetta@gmail.com (S.D.S.); claudio.ferrante@unich.it (C.F.)

3 Laboratoire de Botanique et Phytothérapie, Unité de Formation et de Recherche Sciences de la Nature, 02 BP 801 Abidjan 02, Université Nangui Abrogoua, 00225 Abidjan, Côte d'Ivoire; kouadio777@gmail.com

4 Agricultural and Molecular Research and Service Institute, University of Nyíregyháza, 4400 Nyíregyháza, Hungary; cziaky.zoltan@nye.hu (Z.C.); jjozsi@gmail.com (J.J.)

5 Institute of Research and Development, Duy Tan University, Da Nang 550000, Vietnam; f.mahomoodally@uom.ac.mu

6 Department of Health Sciences, Faculty of Science, University of Mauritius, 230 Réduit, Mauritius; picotcarene@yahoo.com

* Correspondence: giustino.orlando@unich.it (G.O.); gokhanzengin@selcuk.edu.tr (G.Z.)

Received: 1 March 2020; Accepted: 26 March 2020; Published: date

Chemical composition

Chemical compositions of the *B. abyssinica* extracts were determined using a Dionex Ultimate 3000RS UHPLC instrument. The extracts were filtered through 0.22 µm PTFE syringe filter (Labex Ltd, Hungary) before HPLC analysis. The compounds were separated on a Thermo Accucore C18 (100 mm x 2.1, mm i. d., 2.6 µm) column thermostated at 25 °C (± 1 °C). The solvents used were water (A) and methanol (B), both were acidified with 0.1 % formic acid. The flow rate was maintained at 0.2 mL min^{-1}. The elution gradient was isocratic 5 % B (0-3 min), a linear gradient increasing from 5% B to 100% (3-43 min), 100% B (43-61 min), a linear gradient decreasing from 100% B to 5% (61-62 min) and 5% B (62-70 min). The column was coupled to a Thermo Q Exactive Orbitrap mass spectrometer (Thermo Scientific, USA) equipped with electrospray ionization source. MS spectra were recorded in positive and negative-ion mode, respectively.

Trace Finder 3.1 (Thermo Scientific, USA) software was applied for target screening. The compounds listed in the tables were identified on the basis our previous published works or data found in literature using exact molecular mass, isotopic pattern and characteristic fragment ions. In every case, the exact molecular mass, isotopic pattern, characteristic fragment ions and retention time (min) were used for the identification of the compounds which are marked that were confirmed by standards.

Table S1. Chemical composition of ethyl acetate extract.

No.	Name	Formula	Rt (min)	$[M + H]^+$	$[M - H]^-$	Fragment 1	Fragment 2	Fragment 3	Fragment 4	Fragment 5	Literature
1	Trigonelline	C7H8NO2	1,22	138,05550		110,0606	96,0450	94,0657	92,0501		
2[1]	Catechin	C15H14O6	13,28		289,07121	245,0818	203,0697	151,0389	125,0230	109,0281	
3[1]	Vanillin	C8H8O3	15,47	153,05517		125,0601	111,0446	110,0368	93,0341	65,0394	
4	Loliolide	C11H16O3	19,45	197,11777		179,1071	161,0964	135,1172	133,1016	107,0861	
5	Isoliquiritigenin	C15H12O4	24,72		255,06574	153,0177	135,0076	119,0489	91,0174		
6[1]	Eriodictyol (3′,4′,5,7-Tetrahydroxyflavanone)	C15H12O6	24,85		287,05556	151,0026	135,0441	125,0232	107,0126	83,0123	
7	Di-O-methylellagic acid-O-pentoside	C21H18O12	25,31		461,07201	446,0487	328,0227	312,9990	297,9755		
8	Methoxy-pentahydroxy(iso)flavone	C16H12O8	25,74		331,04540	316,0228	287,0201	271,0251	259,0258		
9	Dihydroxyflavone	C15H10O4	26,78		253,05008	208,0523	135,0076	133,0284	117,0332	91,0174	
10	Dihydroxy-dimethoxy(iso)flavone isomer 1	C17H14O6	26,82		313,07122	298,0494	270,0528	269,0450	255,0303		
11[1]	Naringenin (4',5,7-Trihydroxyflavanone)	C15H12O5	27,16		271,06065	227,0707	177,0182	151,0026	119,0489	107,0126	
12	Dihydroxy-dimethoxy(iso)flavone isomer 2	C17H14O6	27,66		313,07122	298,0489	270,0523	269,0472	255,0303	227,0345	
13[1]	Luteolin (3',4',5,7-Tetrahydroxyflavone)	C15H10O6	27,81		285,03991	217,0504	199,0398	175,0393	151,0026	133,0283	
14	3,3'-Di-O-methylellagic acid	C16H10O8	27,84		329,02975	314,0073	298,9840	270,9884			
15	Methoxy-tetrahydroxy(iso)flavone	C16H12O7	28,18		315,05048	300,0279	271,0252	255,0300			
16	Dimethoxy-tetrahydroxy(iso)flavone	C17H14O8	28,45		345,06105	330,0392	315,0152	287,0201			
17	Dihydroxy-dimethoxy(iso)flavone isomer 3	C17H14O6	29,15		313,07122	298,0487	283,0261	255,0293			
18[1]	Apigenin (4',5,7-Trihydroxyflavone)	C15H10O5	29,62		269,04500	227,0344	225,0552	151,0026	149,0233	117,0331	
19	Chrysoeriol (Scoparol, 3'-Methoxy-4',5,7-trihydroxyflavone)	C16H12O6	29,84		299,05556	284,0330	256,0377	227,0346	151,0023		
20	Liquiritigenin (4',7-Dihydroxyflavanone)	C15H12O4	29,92		255,06574	153,0182	135,0076	119,0489	91,0174		
21	Methoxy-trihydroxy(iso)flavone isomer 1	C16H12O6	30,29		299,05556	284,0331	256,0382	255,0299	227,0346		
22	Dimethoxy-trihydroxy(iso)flavone isomer 1	C17H14O7	30,50		329,06613	314,0436	299,0198	285,0407	271,0252	243,0297	
23	Dihydroxy-dimethoxy(iso)flavone isomer 4	C17H14O6	31,18		313,07122	298,0490	297,0406	283,0254	255,0295		
24	3,3',4-Tri-O-methylflavellagic acid	C17H12O9	31,20		359,04031	344,0174	328,9946	313,9712	300,9995	285,9760	
25	Dihydroxy-trimethoxy(iso)flavone isomer 1	C18H16O7	31,77		343,08178	328,0594	313,0361	298,0129	285,0418	270,0164	
26	Methoxy-trihydroxy(iso)flavone isomer 2	C16H12O6	32,45		299,05556	284,0330	256,0371	227,0350	133,0282		
27	Dihydroxy-trimethoxy(iso)flavone isomer 2	C18H16O7	32,50		343,08178	328,0603	313,0355	285,0410	270,0177		
28	Dimethoxy-trihydroxy(iso)flavone isomer 2	C17H14O7	32,71		329,06613	314,0441	299,0202	285,0409	271,0252		
29	Dihydroxy-trimethoxy(iso)flavone isomer 3	C18H16O7	33,09		343,08178	328,0588	313,0358	298,0124	285,0418	270,0171	

30	Dihydroxy-dimethoxy(iso)flavone isomer 5	C17H14O6	34,33		313,07122	298,0488	283,0252	255,0298			
31	Dihydroxy-trimethoxy(iso)flavone isomer 4	C18H16O7	34,54		343,08178	328,0594	313,0371	285,0407			
32	Dihydroxy-dimethoxy(iso)flavone isomer 6	C17H14O6	34,87		313,07122	298,0485	283,0255	255,0310			
33	Dihydroxy-trimethoxy(iso)flavone isomer 5	C18H16O7	34,90		343,08178	328,0590	313,0359	285,0408	269,0461		
34	Bruguierol A	C12H14O2	36,05	191,10721		173,0965	161,0960	147,0807	135,0809	107,0497	
35	Dihydropiptadenin or isomer	C30H48O5	39,38		487,34236	469,3324	443,3537	425,3432	409,3114		
36	Hexadecanedioic acid	C16H30O4	40,29		285,20659	267,1970	223,2063				
37	Piptadenin	C30H46O5	43,40		485,32670	467,3198	441,3390	423,3278	407,2950		(Dawe et al., 2017)
38	Tetrahydropiptadenin or isomer	C30H50O5	43,47		489,35801	471,3493	445,3696	427,3584	411,3273		
39	Hydroxyhexadecanoic acid	C16H32O3	44,87		271,22732	253,2172	225,2221	223,2067			
40	22β-Hydroxyoleanic acid	C30H48O4	47,51		471,34743	453,3373	427,3589	409,3481	393,3168		(Dawe et al., 2017)
41	5α-Stigmast-7,22-dien-3-one	C29H46O	52,03	411,36269		393,3540	327,2687	109,0654	97,0655	83,0862	(Dawe et al., 2017)
42	β-Sitostenone	C29H48O	52,73	413,37835		395,3688	255,2114	123,0809	109,0654	97,0655	

Table S2. Chemical composition of methanol extract.

No.	Name	Formula	Rt (min)	$[M + H]^+$	$[M - H]^-$	Fragment 1	Fragment 2	Fragment 3	Fragment 4	Fragment 5	Literature
1	Trigonelline	C7H8NO2	1,20	138,05550		110,0606	96,0450	94,0658	92,0501		
2	Gallocatechin (Casuarin, Gallocatechol)	C15H14O7	4,56		305,06613	261,0775	219,0660	167,0339	137,0234	125,0231	
3[1]	Catechin	C15H14O6	13,26		289,07121	245,0819	203,0709	151,0390	125,0231	109,0281	
4[1]	Epigallocatechin (Epigallocatechol)	C15H14O7	13,58		305,06613	261,0771	219,0665	167,0340	137,0233	125,0231	
5[1]	Vanillin	C8H8O3	15,47	153,05517		125,0601	111,0446	110,0368	93,0341	65,0393	
6	Naringenin-6,8-di-C-glucoside	C27H32O15	16,90		595,16630	577,1545	505,1340	475,1252	385,0933	355,0827	
7	Quercetin-O-hexoside	C21H20O12	19,15		463,08765	301,0369	300,0280	271,0253	255,0299	243,0294	
8	Loliolide	C11H16O3	19,47	197,11777		179,1071	161,0964	135,1173	133,1016	107,0861	
9	Trihydroxystilbene	C14H12O3	19,66	229,08647		183,0809	165,0704	135,0446	119,0497	107,0497	
10	Tetrahydroxyxanthone	C13H8O6	21,97		259,02427	231,0292	215,0352	203,0342	187,0396		
11	Isoliquiritigenin	C15H12O4	24,74		255,06574	153,0183	135,0076	119,0489	91,0175		
12[1]	Eriodictyol (3′,4′,5,7-Tetrahydroxyflavanone)	C15H12O6	24,87		287,05556	151,0026	135,0440	125,0231	107,0125	83,0122	
13	Abscisic acid	C15H20O4	25,34		263,12834	219,1386	204,1151	201,1284	152,0834	151,0753	
14	Methoxy-pentahydroxy(iso)flavone	C16H12O8	25,75		331,04540	316,0224	287,0197	271,0255	259,0258		
15	Dihydroxy-methoxy(iso)flavone isomer 1	C16H12O5	26,48		283,06065	268,0380	267,0304	240,0426			
16	Dihydroxyflavone	C15H10O4	26,79		253,05008	208,0524	135,0076	133,0284	117,0332	91,0174	
17	Dihydroxy-dimethoxy(iso)flavone isomer 1	C17H14O6	26,83		313,07122	298,0494	270,0528	269,0450	255,0303		
18[1]	Quercetin (3,3',4',5,7-Pentahydroxyflavone)	C15H10O7	26,95		301,03483	273,0412	178,9975	151,0026	121,0281	107,0124	
19	Methoxy-tetrahydroxy(iso)flavone-O-hexoside	C22H22O12	27,07		477,10331	315,0518	314,0439	299,0200	271,0250		
20[1]	Naringenin (4',5,7-Trihydroxyflavanone)	C15H12O5	27,17		271,06065	227,0716	177,0183	151,0025	119,0489	107,0125	
21	Homoeriodictyol (3'-Methoxy-4',5,7-trihydroxyflavanone)	C16H14O6	27,25		301,07122	196,0009	177,0183	151,0026	149,0597	134,0362	

22	Dihydroxy-dimethoxy(iso)flavone isomer 2	C17H14O6	27,67		313,07122	298,0485	270,0537	269,0468	255,0299	227,0344
23[1]	Luteolin (3',4',5,7-Tetrahydroxyflavone)	C15H10O6	27,82		285,03991	217,0501	199,0395	175,0391	151,0026	133,0283
24	Methoxy-tetrahydroxy(iso)flavone	C16H12O7	28,20		315,05048	300,0278	271,0251	255,0300		
25	Dimethoxy-tetrahydroxy(iso)flavone	C17H14O8	28,45		345,06105	330,0392	315,0152	287,0201		
26	Dihydroxy-dimethoxy(iso)flavone isomer 3	C17H14O6	29,16		313,07122	298,0486	283,0254	255,0301		
27	Dimethoxy-trihydroxy(iso)flavone-O-hexoside	C23H24O12	29,55		491,11895	329,0671	314,0436	299,0199	271,0245	
28[1]	Apigenin (4',5,7-Trihydroxyflavone)	C15H10O5	29,63		269,04500	227,0351	225,0552	151,0026	149,0233	117,0331
29	Chrysoeriol (Scoparol, 3'-Methoxy-4',5,7-trihydroxyflavone)	C16H12O6	29,86		299,05556	284,0330	256,0376	227,0344	151,0031	
30	Liquiritigenin (4',7-Dihydroxyflavanone)	C15H12O4	29,93		255,06574	153,0182	135,0076	119,0489	91,0174	
31	Methoxy-trihydroxy(iso)flavone isomer 1	C16H12O6	30,31		299,05556	284,0330	256,0377	255,0299	227,0346	
32	Dimethoxy-trihydroxy(iso)flavone isomer 1	C17H14O7	30,52		329,06613	314,0437	299,0202	285,0410	271,0253	243,0298
33	Dihydroxy-dimethoxy(iso)flavone isomer 4	C17H14O6	31,21		313,07122	298,0492	297,0407	283,0254	255,0296	
34	Dihydroxy-trimethoxy(iso)flavone isomer 1	C18H16O7	31,77		343,08178	328,0613	313,0359	298,0127	285,0418	270,0161
35	Dihydroxy-tetramethoxy(iso)flavone	C19H18O8	32,42	375,10800		360,0845	345,0606	317,0658		
36	Methoxy-trihydroxy(iso)flavone isomer 2	C16H12O6	32,48		299,05556	284,0333	256,0368	227,0351	133,0284	
37	Dihydroxy-trimethoxy(iso)flavone isomer 2	C18H16O7	32,52		343,08178	328,0600	313,0357	285,0406	270,0171	
38	Dimethoxy-trihydroxy(iso)flavone isomer 2	C17H14O7	32,72		329,06613	314,0437	299,0202	285,0403	271,0251	
39	Dihydroxy-trimethoxy(iso)flavone isomer 3	C18H16O7	33,10		343,08178	328,0585	313,0353	298,0123	285,0418	270,0170
40	Dihydroxy-dimethoxy(iso)flavone isomer 5	C17H14O6	34,36		313,07122	298,0487	283,0250	255,0299		
41	Dihydroxy-methoxy(iso)flavone isomer 2	C16H12O5	34,46		283,06065	268,0380	267,0289	240,0433		
42	Dihydroxy-trimethoxy(iso)flavone isomer 4	C18H16O7	34,56		343,08178	328,0598	313,0359	285,0408		
43	Dihydroxy-dimethoxy(iso)flavone isomer 6	C17H14O6	34,87		313,07122	298,0487	283,0251	255,0303		
44	Dihydroxy-trimethoxy(iso)flavone isomer 5	C18H16O7	34,90		343,08178	328,0597	313,0359	285,0410	269,0455	
45	Bruguierol A	C12H14O2	36,04	191,10721		173,0960	161,0964	147,0808	135,0809	107,0497

46	Dihydropiptadenin or isomer	C30H48O5	39,39		487,34236	469,3331	443,3549	425,3440	409,3122		
47	Hexadecanedioic acid	C16H30O4	40,29		285,20659	267,1966	223,2065				
48	Piptadenin	C30H46O5	43,40		485,32670	467,3201	441,3401	423,3274	407,2947		(Dawe et al., 2017)
49	Tetrahydropiptadenin or isomer	C30H50O5	43,47		489,35801	471,3476	445,3690	427,3585	411,3275		
50	Hydroxyhexadecanoic acid	C16H32O3	44,87		271,22732	253,2189	225,2220	223,2067			
51	22β-Hydroxyoleanic acid	C30H48O4	47,51		471,34743	453,3384	427,3583	409,3484	393,3172		(Dawe et al., 2017)
52	5α-Stigmast-7,22-dien-3-one	C29H46O	52,04	411,36269		393,3542	327,2664	109,0653	97,0654	83,0862	(Dawe et al., 2017)
53	β-Sitostenone	C29H48O	52,71	413,37835		395,3675	255,2116	123,0807	109,0654	97,0655	

Table S3. Chemical composition of water extract.

No.	Name	Formula	Rt (min)	[M + H]$^+$	[M - H]$^-$	Fragment 1	Fragment 2	Fragment 3	Fragment 4	Fragment 5
1	Trigonelline	C7H8NO2	1,24	138,05550		110,0604	96,0450	94,0657	92,0500	
2	Gallocatechin (Casuarin, Gallocatechol)	C15H14O7	4,42		305,06613	261,0761	219,0651	167,0337	137,0229	125,0227
3[1]	Catechin	C15H14O6	13,18		289,07121	245,0811	203,0705	151,0385	125,0228	109,0279
4[1]	Epigallocatechin (Epigallocatechol)	C15H14O7	13,50		305,06613	261,0760	219,0659	167,0335	137,0228	125,0227
5[1]	Vanillin	C8H8O3	15,57	153,05517		125,0601	111,0445	110,0367	93,0341	65,0394
6	Naringenin-6,8-di-C-glucoside	C27H32O15	16,87		595,16630	577,1549	505,1356	475,1251	385,0930	355,0819
7	Quercetin-O-hexoside	C21H20O12	19,10		463,08765	301,0348	300,0270	271,0249	255,0295	243,0288
8	Loliolide	C11H16O3	19,57	197,11777		179,1070	161,0963	135,1172	133,1016	107,0860
9	Tetrahydroxyxanthone	C13H8O6	21,92		259,02427	231,0292	215,0344	203,0338	187,0390	
10	Isoliquiritigenin	C15H12O4	24,66		255,06574	153,0179	135,0073	119,0486	91,0172	
11[1]	Eriodictyol (3′,4′,5,7-Tetrahydroxyflavanone)	C15H12O6	24,79		287,05556	151,0022	135,0437	125,0227	107,0123	83,0120
12	Abscisic acid	C15H20O4	25,26		263,12834	219,1380	204,1145	201,1277	152,0828	151,0750
13	Methoxy-pentahydroxy(iso)flavone	C16H12O8	25,70		331,04540	316,0212	287,0195	271,0251	259,0258	
14	Dihydroxy-methoxy(iso)flavone isomer 1	C16H12O5	26,41		283,06065	268,0376	267,0291	240,0417		
15	Dihydroxyflavone	C15H10O4	26,71		253,05008	208,0522	135,0074	133,0283	117,0329	91,0173
16	Dihydroxy-dimethoxy(iso)flavone isomer 1	C17H14O6	26,75		313,07122	298,0480	270,0526	269,0443	255,0322	
17[1]	Quercetin (3,3',4',5,7-Pentahydroxyflavone)	C15H10O7	26,87		301,03483	273,0389	178,9974	151,0022	121,0280	107,0121
18	Methoxy-tetrahydroxy(iso)flavone-O-hexoside	C22H22O12	27,01		477,10331	315,0498	314,0430	299,0195	271,0245	
19[1]	Naringenin (4',5,7-Trihydroxyflavanone)	C15H12O5	27,08		271,06065	227,0711	177,0179	151,0022	119,0486	107,0123
20	Homoeriodictyol (3'-Methoxy-4',5,7-trihydroxyflavanone)	C16H14O6	27,18		301,07122	196,0001	177,0173	151,0022	149,0593	134,0358
21	Dihydroxy-dimethoxy(iso)flavone isomer 2	C17H14O6	27,62		313,07122	298,0491	270,0537	269,0437	255,0287	227,0339

22[1]	Luteolin (3',4',5,7-Tetrahydroxyflavone)	C15H10O6	27,74	285,03991	217,0495	199,0393	175,0385	151,0023	133,0279
23	Methoxy-tetrahydroxy(iso)flavone	C16H12O7	28,13	315,05048	300,0272	271,0245	255,0291		
24	Dimethoxy-trihydroxy(iso)flavone-O-hexoside	C23H24O12	29,48	491,11895	329,0665	314,0431	299,0197	271,0249	
25	Chrysoeriol (Scoparol, 3'-Methoxy-4',5,7-trihydroxyflavone)	C16H12O6	29,77	299,05556	284,0321	256,0373	227,0344	151,0030	
26	Liquiritigenin (4',7-Dihydroxyflavanone)	C15H12O4	29,86	255,06574	153,0176	135,0069	119,0486	91,0172	
27	Methoxy-trihydroxy(iso)flavone isomer 1	C16H12O6	30,22	299,05556	284,0324	256,0376	255,0295	227,0340	
28	Dimethoxy-trihydroxy(iso)flavone isomer 1	C17H14O7	30,44	329,06613	314,0430	299,0196	285,0397	271,0249	243,0294
29	Methoxy-trihydroxy(iso)flavone isomer 2	C16H12O6	32,39	299,05556	284,0323	256,0368	227,0350	133,0281	
30	Dimethoxy-trihydroxy(iso)flavone isomer 2	C17H14O7	32,63	329,06613	314,0432	299,0195	285,0400	271,0242	
31	Dihydroxy-methoxy(iso)flavone isomer 2	C16H12O5	34,35	283,06065	268,0373	267,0286	240,0432		
32	Emodin	C15H10O5	38,90	269,04500	241,0496	225,0549			
33	Dihydropiptadenin or isomer	C30H48O5	39,30	487,34236	469,3330	443,3508	425,3429	409,3126	
34	Hexadecanedioic acid	C16H30O4	40,22	285,20659	267,1959	223,2055			
35	Tetrahydropiptadenin or isomer	C30H50O5	43,40	489,35801	471,3472	445,3681	427,3573	411,3254	
36	Hydroxyhexadecanoic acid	C16H32O3	44,80	271,22732	253,2183	225,2215	223,2062		

[1] Confirmed by standard

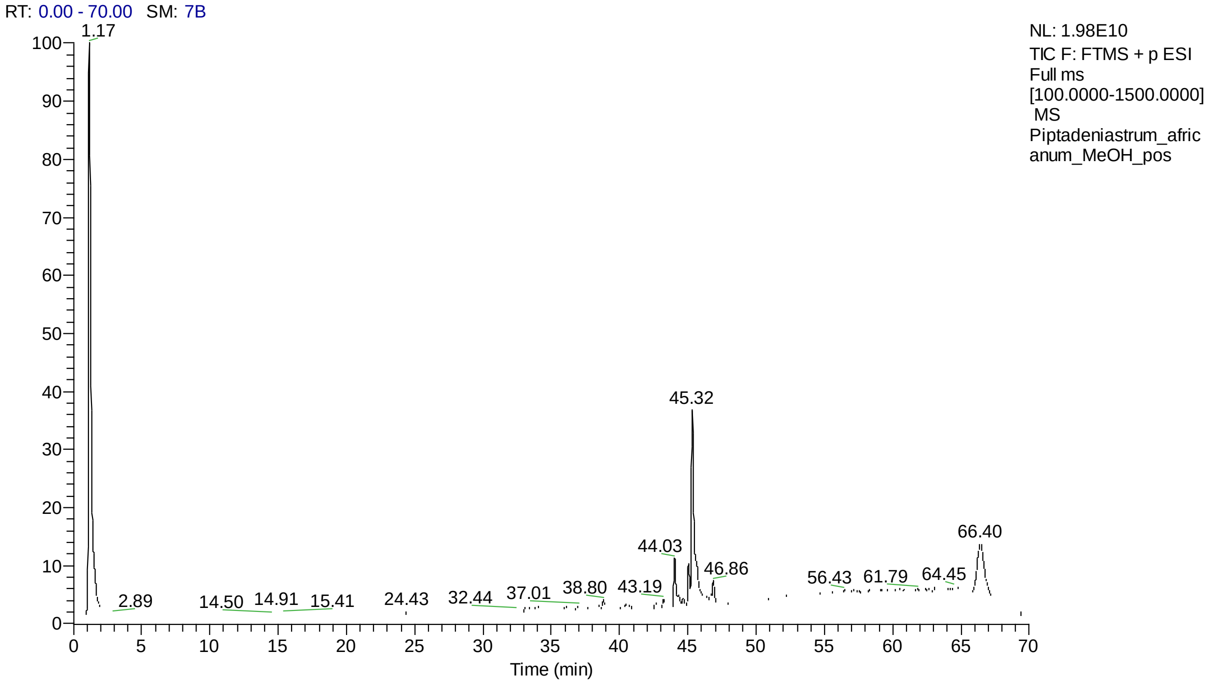

(a)

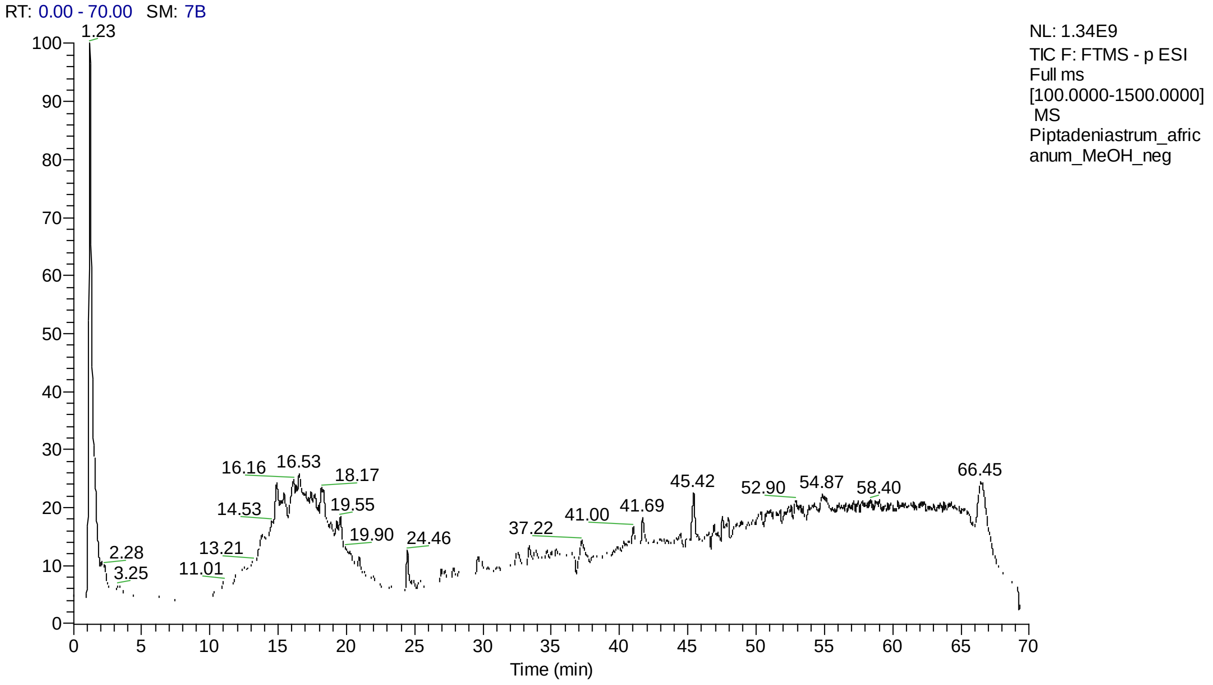

(b)

Figure S1. Total ion chromatogram of methanol extract in positive (a) and negative (b) ion mode

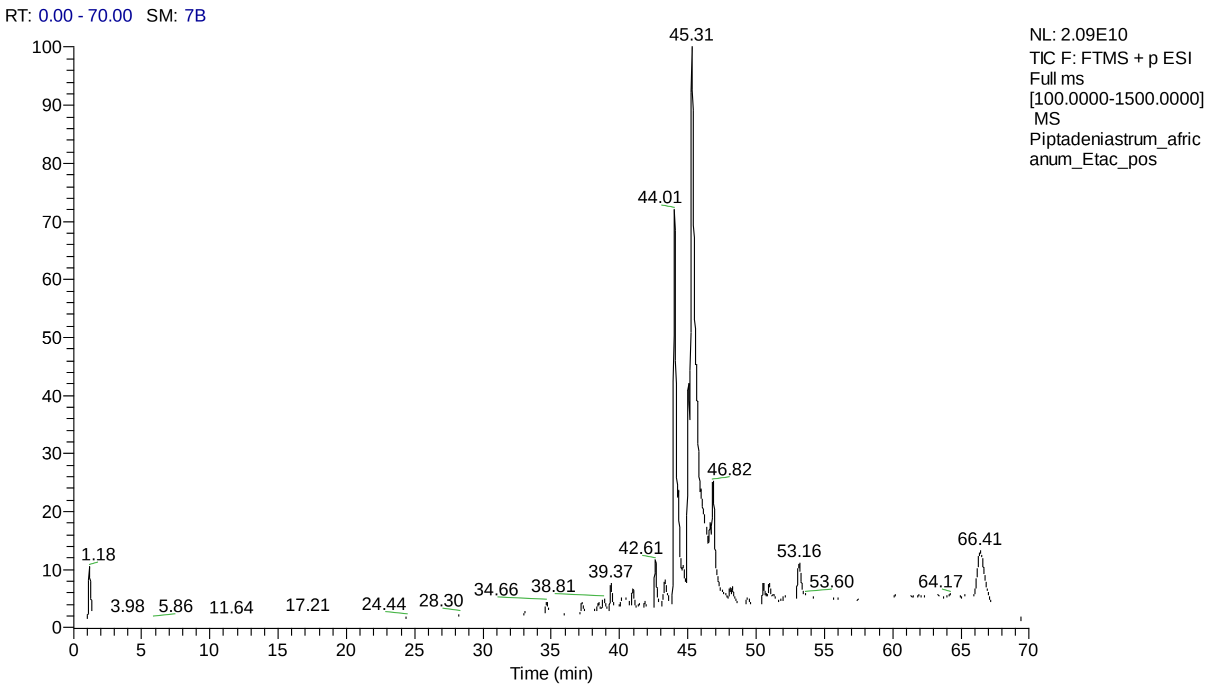

(a)

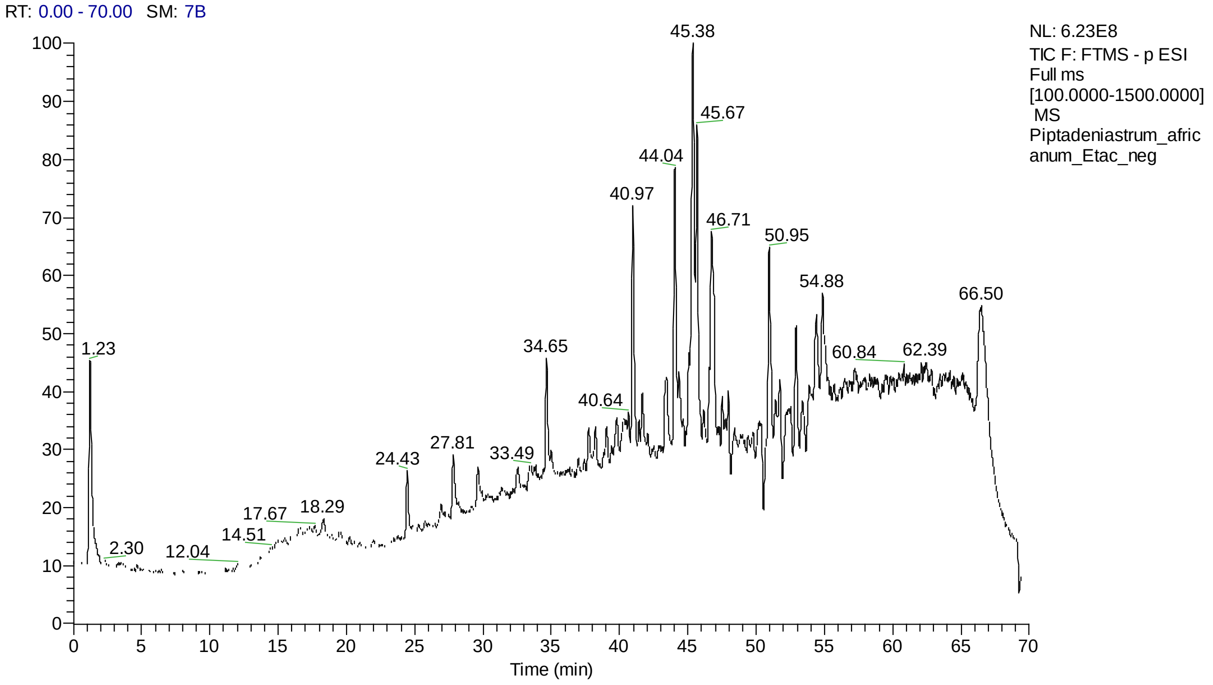

(b)

Figure S2. Total ion chromatogram of ethyl acetate extract in positive (a) and negative (b) ion mode

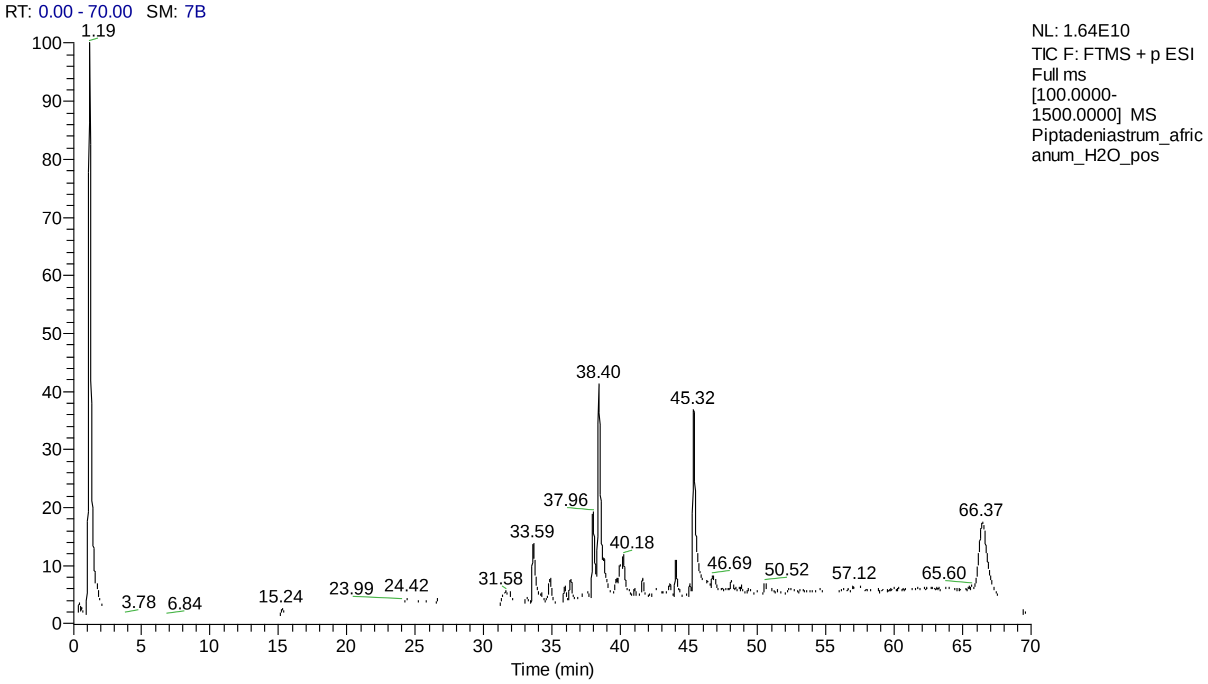

(a)

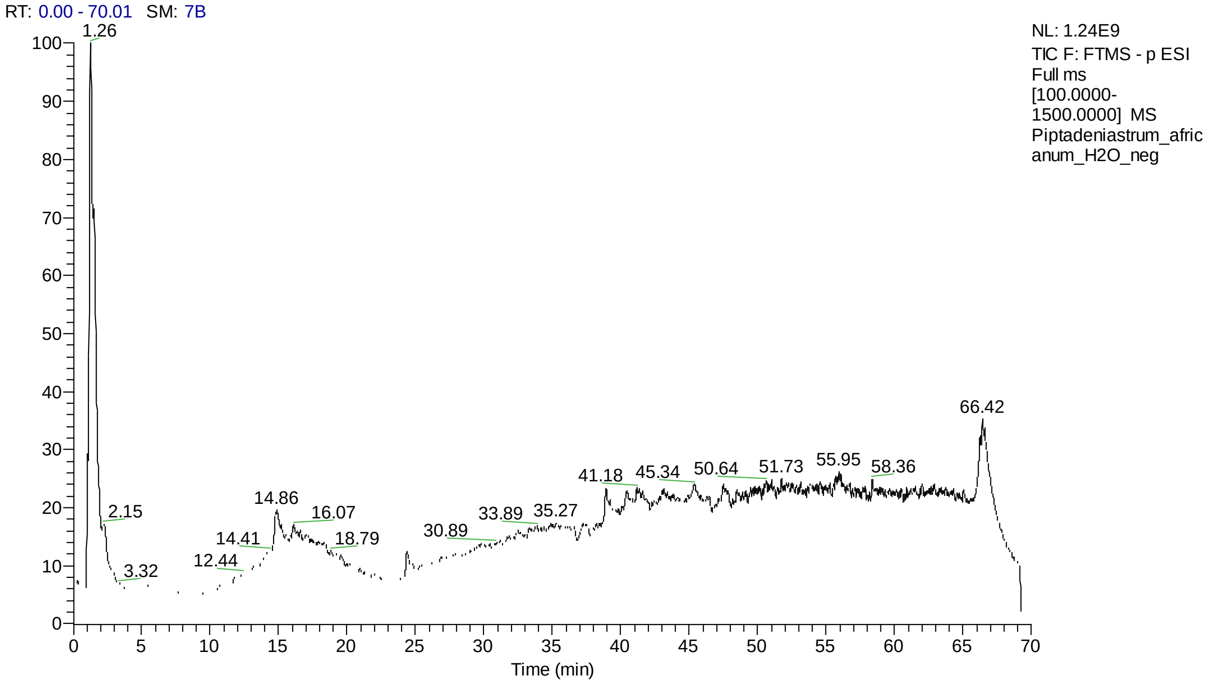

(b)

Figure S3. Total ion chromatogram of water extract in positive (a) and negative (b) ion mode
